# Contributions of T-helper 9 cells in endometriosis-associated inflammation and lesion growth

**DOI:** 10.1093/jimmun/vkag123

**Published:** 2026-05-29

**Authors:** Alison McCallion, Danielle J Sisnett, Katherine B Zutautas, Priyanka Yolmo, Harshavardhan Lingegowda, Kasthuri Ravishanker, Dan Vo Hoang, Chandrakant Tayade

**Affiliations:** Department of Biomedical and Molecular Sciences, Queen’s University, Kingston, ON, Canada; Department of Biomedical and Molecular Sciences, Queen’s University, Kingston, ON, Canada; Department of Biomedical and Molecular Sciences, Queen’s University, Kingston, ON, Canada; Department of Biomedical and Molecular Sciences, Queen’s University, Kingston, ON, Canada; Department of Biomedical and Molecular Sciences, Queen’s University, Kingston, ON, Canada; Department of Biomedical and Molecular Sciences, Queen’s University, Kingston, ON, Canada; Department of Biomedical and Molecular Sciences, Queen’s University, Kingston, ON, Canada; Department of Biomedical and Molecular Sciences, Queen’s University, Kingston, ON, Canada

**Keywords:** endometriosis, estrogen, inflammation, T-helper 9 cells

## Abstract

Endometriosis is an inflammatory gynecologic disease characterized by ectopic growth of endometrial-like tissue, resulting in pelvic pain and infertility. T-helper 9 (Th9) cells play a known role in various chronic inflammatory diseases. Despite parallels between endometriosis and Th9-driven diseases, their role in endometriosis has not been extensively explored. We investigated Th9 cell involvement in endometriosis pathophysiology using human tissue samples, in vitro experiments with human-derived Th9 cells, and in vivo experiments to shed insight on the impact of adoptively transferred Th9 cells in our established syngeneic endometriosis mouse model. Immunohistochemistry of a tissue microarray revealed significantly increased IL-9–positive cells in patient lesions compared to control endometrium. Human CD4^+^ Th cells purified from peripheral blood mononuclear cells treated with Th9-driving growth factors produced significantly altered proinflammatory mediators (increased IL-5 and IL-17F; decreased IL-8) in response to estrogen stimulation. Adoptive transfer of mouse Th9-like cells increased plasma IL-1α concentration and altered transcriptional profiles of several signaling pathways, including Notch and PI3K-Akt. Immunofluorescent microscopy depicted adoptively transferred Th9 cells present within mouse lesions. Furthermore, immunohistochemical analysis demonstrated reduced lesion proliferation following Th9 adoptive transfer. This study provides the first evidence that Th9 cells likely promote immune-inflammatory alterations within lesions to exacerbate disease.

## Introduction

Endometriosis (EMS) is a chronic inflammatory disease wherein endometrial-like (endometriotic) tissue proliferates at ectopic sites, most commonly on peritoneal surfaces and pelvic organs. Our group and others have provided evidence that immune dysregulation in EMS is a predominant mechanism associated with disease progression.^1–4^ This, combined with an estrogen-dominant, progesterone-resistant endocrine imbalance, further shapes the immune landscape.

T-helper type 9 (Th9) cells are a specialized Th subset named for their production of interleukin 9 (IL-9). These cells are involved in allergic responses and inflammation in parasitic infections, parallel to the classical roles of mast cells (MCs). Th9 cells differentiate from naïve CD4^+^ T cells when exposed to IL-4 and TGF-β, but can also differentiate from Th2 cells when stimulated with TGF-β.^5^^,^^6^ While its function and regulation are still not fully understood, IL-9 is known as a pleiotropic cytokine with roles in T-cell and MC development, and has been implicated in numerous pathologies as a regulator of inflammatory and proliferative signals.^7^^,^^8^ In addition, IL-9 has been identified for its role in fibrosis of the lungs and airway in subepithelial compartments,^9^ and was reported as a reliable marker of poor healing in ulcerative colitis.^10^

Evidence in literature indicates that IL-9 signaling is elevated in EMS. In 2012, Lessey et al. documented increased concentrations of IL-9 in the peritoneal fluid (PF) of patients with EMS.^11^ We also previously demonstrated that IL-9 levels were significantly higher in plasma of patients compared to fertile healthy controls, and that EMS lesions as well as matched patient eutopic endometrium produced IL-9.^12^ More recently, Tarumi et al. identified a significantly higher presence of IL-9^+^CD4^+^ immune cells in the PF of patients with EMS compared to controls.^13^

The dysregulation of several Th subtype populations has been documented in EMS pathophysiology.^4^ Our group recently demonstrated the dysregulated functioning of Th17 cells in EMS,^14^ and the disease’s overall immune dysregulation is known to skew toward Th2 responses.^2^ Furthermore, we have previously reported on the involvement of MCs in EMS,^15^ a cell type that relies on IL-9 for development. However, the involvement of Th9 cells and IL-9 in EMS has not yet been explored. Th9 cells have been identified to have important roles in allergic and autoimmune diseases, cystic fibrosis, and several cancers including endometrial carcinoma, where IL-9 was regulated by progesterone receptor expression.^8^^,^^16^

Inflammation and proliferation are central to EMS lesion progression, and the alteration of these processes by immune dysregulation is continually being investigated. Here, we address the knowledge gap surrounding the potential role of Th9 cells in modulating endometriotic lesion–associated immune inflammatory alterations. Our study identifies the elevated presence of IL-9 in human EMS lesions, examines the effects of estradiol (E2) and progesterone (P4) on the cytokine secretory profile of Th9-driven human T cells, and evaluates the impacts of Th9 cell adoptive transfer in a mouse model of EMS.

## Materials and methods

### Study approval

Human lesion samples were obtained with written informed consent from patients who underwent laparoscopic excision surgery and/or hysterectomy at Kingston General Hospital, Kingston, ON, Canada. The endometrial samples, both from patients and healthy individuals matched for uterine cycle phase, were obtained by pipelle sampling, as per standard procedure. Blood was collected from volunteers with informed consent by a certified phlebotomist at the Department of Biomedical Sciences, Queen’s University (Kingston, ON, Canada). The study was approved by the Queen’s University Health Sciences Research Ethics Board. In vivo experiments were done using C57BL/6 mice acquired from Charles River Laboratories and The Jackson Laboratory. Experiments were approved by the Queen’s Institutional Animal Care Committee, Kingston, ON, Canada.

### Tissue microarray of human patient samples

In collaboration with the Department of Pathology at the Kingston Health Sciences Center (Kingston, ON, Canada), a tissue microarray was constructed using triplicate 1-mm cores of ovarian endometriotic lesions, matched eutopic endometrium, and healthy control endometrium.^17^ These samples were collected from a cohort of patients with EMS aged <47 years and undergoing laparoscopic surgery and hysterectomy, and healthy patients <44 years of age who were undergoing hysterectomy surgeries and showed no signs of EMS or other pathologies. At time of staining, integrity of cores was evaluated and excluded if more than 50% of tissue area was missing. After exclusions, there were *n* = 10 ovarian endometriotic lesion samples, and eutopic endometrium samples (*n* = 12) were stratified into secretory (*n* = 5), proliferative (*n* = 5), or inactive endometrium (*n* = 2). Control endometrium samples (*n* = 5) were all in the proliferative phase.

### Evaluating IL-9 presence in tissue microarray of endometrioma lesions, eutopic endometrium, and control endometrium

Immunohistochemistry for anti-IL-9 antibody staining was performed on a human patient tissue microarray of endometrioma lesions, eutopic endometrium, and control endometrium. Triplicates of control endometrium (*n* = 5), eutopic patient endometrium (*n* = 12), and endometrioma tissues (*n* = 10) were stained with anti-IL-9 monoclonal antibody (66144-1-IG, Thermo Fisher Scientific). The tissue microarray was stained using a Leica Bond RX automated stainer (Leica Microsystems). EDTA-based epitope retrieval was conducted for 10 min and primary antibody was incubated for 15 min at a concentration of 1:200. Leica BOND Polymer Refine Detection kit (DS9800, Leica Biosystems) was used for detection of 3,3′-diaminobenzidine (DAB) chromogen against a hematoxylin counterstain (Leica Microsystems). Slides were scanned at 40× magnification in an Olympus VS120 high-resolution slide scanner (Olympus Life Science). Image analysis was performed using HALO AI software (Indica Labs). Scanned images were classified into slide glass (empty area), glandular epithelium, glandular lumen, stroma (proliferative and secretory), and vasculature using HALO’s tissue classifier tool in order to exclude empty areas from analysis. A cytonuclear analysis algorithm was optimized to detect weak, moderate, and strong positive staining of anti-IL-9 antibody. A one-way ANOVA test of grouped data in triplicates was used to compare percentage of positively stained cells out of all detected cells in endometrioma tissues, eutopic endometrium, and control endometrium. Outlier test (ROUT Q = 0.5%) found no outliers.

### Driving Th9 cells from human PBMCs

Human blood was collected by venipuncture and PBMCs were separated by centrifugation using Lymphoprep density gradient medium (18061) in SepMate tubes (85450) as per StemCell Technologies protocol. CD4^+^ T cells were isolated by immunomagnetic negative selection using the EasySep Human Naïve CD4^+^ T Cell Isolation Kit II (17555, StemCell Technologies Canada Inc.). Media for Th9 derivation culture consisted of RPMI 1640 medium (11875093, Thermo Fisher Scientific) with 10% FBS (CA76327-086, VWR), 1% penicillin-streptomycin (15140122, Thermo Fisher Scientific), 1% sodium pyruvate (11360070, Thermo Fisher Scientific), 1% MEM-nonessential amino acids (11140076, Thermo Fisher Scientific), 0.5% HEPES (15630080, Thermo Fisher Scientific), 0.5% L-glutamine (25030081, Thermo Fisher Scientific), and 0.05% β-mercaptoethanol (31350010, Thermo Fisher Scientific). Antibodies and soluble cytokines were acquired from BioLegend. Purified CD4^+^ naïve T cells were seeded at 225,000 cells/mL in anti-CD3ε-coated flasks (317326) with soluble anti-CD28 (302902), soluble anti-IFN-γ (506531) and recombinant growth factors IL-7 (581902), IL-4 (574002), and TGF-β (781802) in 2 phases, adapted from Pham’s protocol^18^ and Bi et al.’s findings regarding the influence of IL-7 on Th9 differentiation.^19^ To evaluate impact of sex steroid hormones E2 and P4 on the cytokine profile, one triplicate of wells received E2 (1 × 10^−6^ M) and one triplicate received P4 (1 × 10^−6^ M). These concentrations represent physiologically high levels seen in EMS, as demonstrated in our previous work.^15^ Powder forms of E2 (3301, Millipore Sigma) and P4 (P0130, Millipore Sigma) were dissolved in 100% ethanol per manufacturer instructions and diluted to working concentrations in RPMI. As a control, one triplicate of wells had CD4^+^ cells cultured only with anti-CD3ε and anti-CD28 as “Th0” without stimulation of any growth factors. After 5 days, cells were stimulated with phorbol myristate acetate (PMA; 74042, StemCell Technologies Canada, Inc.) and ionomycin (73722, StemCell Technologies Canada, Inc.) for 5 h (or PBS for unstimulated cells) before collecting cells and supernatant. Supernatant was collected and sent for multiplex cytokine analysis (panel HD-32, Eve Technologies, Calgary, AB, Canada).

### Syngeneic mouse model of endometriosis

In vivo experiments were done using our established syngeneic mouse model of EMS.^17^^,^^20^ C57BL/6 mice (*n* = 10) were acquired from Charles River Laboratories (strain code 027) and housed in cages of 4 to 5 mice. Experiments were approved by the Queen’s Institutional Animal Care Committee. Donor mice were euthanized with inhalation of 5% isoflurane for 3 min followed by cervical dislocation. Uterine horns were removed and placed in PBS. Using a dermal biopsy punch, 3-mm^3^ fragments of endometrial tissue were taken and kept on ice in PBS until surgically implanted in recipient mice. Prior to surgery, recipient mice were anesthetized in a vaporizer with 3.5% isoflurane. A small incision was made in the abdomen of each recipient mouse and two 3-mm^3^ fragments of donor mouse endometrium were grafted in the left side of the peritoneal cavity of recipient mice using VetBond adhesive (1469SB, 3M), while sham surgeries involved abdominal incision and suture without implantation of fragments. Postoperative fluid therapy and analgesics were given for 3 days following surgery and lesions were allowed to establish for 14 days. Peritoneal lavage, spleen, and endometriotic lesions were harvested at endpoint.

### Evaluating Th9 presence in mouse model of endometriosis

To capture baseline presence of Th9 cells in a mouse model of EMS, mice were induced with EMS (*n* = 3) or received sham operation (*n* = 3). Endometriotic lesions were allowed to develop over 14 days, then animals were euthanized to harvest spleen and peritoneal immune cells by peritoneal lavage. Flow cytometry was performed in 2 panels of markers to quantify Th9 cell and, separately, MC and committed MC progenitor (MCcp) presence in mouse splenocyte and peritoneal immune cell populations of mice induced with EMS compared to sham-operated mice. Spleens were mechanically digested through a 70-µm strainer into a single-cell suspension in RPMI culture medium (11835030, Thermo Fisher Scientific) with 10% FBS. Peritoneal cells and splenocytes were counted by trypan blue exclusion in a Countess 3 FL automated cell counter (Thermo Fisher Scientific). To detect Th9 cells, peritoneal and spleen cells were stained with fluorescent antibody markers CD45–Pacific Blue, CD4–APC, IL-4Rα–PE/Cy7, and IRF4–PerCP/Cy5.5, as well as CD8α–FITC, CD14–FITC, CD19–FITC, and Zombie Aqua fixable viability dye (BioLegend). Cells that were CD45^+^CD4^+^IL-4Ra^+^IRF4^+^CD8a^−^CD14^−^CD19^−^ were considered Th9 cells. It is important to note that Th9 transcriptional programming involves multiple cooperating factors, including IRF4 and PU.1. As IRF4 alone can activate *IL9* transcription,^21^ and Th9 identity is not exclusively defined by PU.1, only IRF4 was used to characterize Th9 cells in this study. Thus, future studies should aim to also characterize PU.1 expression under these conditions. In the second panel, to detect MC and MCcp, peritoneal cells were stained with fluorescent antibody markers CD45–Pacific Blue, FCERIα–PE, CD117–Brilliant Violet 650, CD11b–FITC, integrin β-7–APC, IL-9R–PE/Cy7, and Zombie Aqua fixable viability dye (BioLegend). MCs were identified as CD45^+^FCERIα^+^CD117^+^CD11b^−^, while MCcps were identified as CD45^+^CD117^+^integrin β-7^+^SSC^lo^. Data for all flow cytometry experiments were acquired using CytoFLEX S (Beckman Coulter) and analyzed via FlowJo v10 software (TreeStar).

### Differentiation of mouse Th9 cells in culture

Spleen and lymph nodes of C57/Bl6 mice (*n* = 3) (Charles River Laboratories) were harvested and mechanically digested through a 70-µm strainer into a single-cell suspension. Naïve CD4^+^ T cells were separated by negative selection using the EasySep Mouse Naïve CD4^+^ T Cell Isolation Kit (19765, StemCell Technologies Canada Inc.) according to manufacturer protocol. Soluble cytokines and antibodies were obtained from BioLegend per the Th9 Polarization Activation Bundle (Th9 Polarization of Mouse CD4^+^ Cells Protocol). Naïve CD4^+^ T cells were cultured in flasks pretreated with plate-bound anti-CD3ε (100340). Cultures were treated with a differentiation cocktail consisting of 5 μg/mL anti-CD28 (102116), 20 ng/mL IL-4 (574302), 2 ng/mL human TGF-β (781802), and 10 μg/mL anti-IFN-γ (505834) for 72 h, followed by 10 ng/mL human IL-2 (575402), 20 ng/mL mouse IL-4, and 1 ng/mL human TGF-β for 48 h in 3× original media volume as per Pham’s published protocol.^18^ One triplicate was treated with 1 × 10^−6^ M E2 (E2758, Sigma Aldrich) to gauge impact of estrogen on Th9 differentiation. At the end of the 48 h phase, CD4^+^ T cells were then activated in vitro with 50 ng/mL PMA and 750 ng/mL ionomycin for 6 h before analysis by flow cytometry. One triplicate was treated with 1 µM monensin-containing protein transport inhibitor GolgiStop (554724, BD Biosciences) to evaluate its impact on phenotype representation after PMA/ionomycin stimulation. Cells were stained with the following antibodies obtained from BioLegend: Pacific Blue–CD45, FITC–CD8α, CD14, CD19, APC–CD4, PerCP/Cy5.5–IRF4, PE-Cy7–IL-4Rα, and Zombie Aqua fixable viability dye. Cells that were CD8α^−^CD14^−^CD19^−^CD4^+^IRF4^+^IL-4Rα^+^ were considered Th9 cells.

### Purification and adoptive transfer of GFP^+^ Th9-like lymphocytes in a mouse model of endometriosis

Spleen and lymph nodes of donor GFP^+^ C57/Bl6 mice (C57BL/6-Tg[UBC-GFP]30Scha/J, strain #004353, Jackson Laboratory) (*n* = 5) were dissected, mechanically digested, and passed through a 70-µm strainer to obtain a single-cell suspension. CD4^+^ cells were isolated and cultured as described above. Before adoptive transfer, Th9 cells were purified by 2 positive selection immunomagnetic separation kits. StemCell EasySep Release Mouse Biotin Positive Selection Kit (17655) and PE Positive Selection Kit (17656) were used per manufacturer protocols to positively select IL-4Rα^+^ and subsequently TGF-β1 RII^+^ cells. As IL-4Rα and TGF-β1 RII are not concurrently expressed in any other T-cell subsets other than Th9 cells, this allowed us to obtain a purified Th9 population. A PE-conjugated TGF-β1 RII antibody (FAB532P, R&D Systems) was used with the PE positive selection kit. A purified IL-4Rα antibody (144801, BioLegend) was biotinylated using Abcam’s biotinylation kit (AB201795) per manufacturer protocol and was used to select IL-4Rα^+^ cells in the biotin positive selection kit. On day 0, EMS was induced in recipient C57/Bl6 mice (*n* = 10) with two 3-mm^3^ endometrial fragments as detailed previously. On day 7, EMS-induced mice (*n* = 5) each received a single intraperitoneal injection of approximately 274,000 GFP^+^, in vitro*–*expanded, purified Th9-like lymphocytes suspended in 100 µL of PBS. Concurrently, a group of EMS-induced mice (*n* = 5) received intraperitoneal injections of 100 µL PBS as a control. Blood was collected at day 7 and day 14 to measure plasma cytokine levels before and after adoptive transfer (Fig. 5A). Specifically, using submandibular vein puncture, 100 µL of blood was collected in EDTA-coated collection tubes and blood was allowed to coagulate on ice for 2 h. Blood samples were centrifuged at 3,000 × *g* for 15 min at 4 °C and separated plasma was aspirated before being diluted 1:2 in preparation for multiplex cytokine analysis. Plasma cytokines, chemokines, and growth factors were analyzed using a commercially available 32-plex panel (MD-32, Eve Technologies, Calgary, AB, Canada). Animals were euthanized on day 14 as previously described in order to collect PF (lavage), lesions, spleen, and uterus. Specifically, one lesion was preserved in freshly prepared 4% paraformaldehyde for 24 h fixation at 4 °C while the other lesion was snap-frozen in liquid nitrogen and stored in −80 °C to be processed for RNA extraction.

### Evaluation of differentially expressed genes using NanoString transcriptomic analysis in endometriotic lesions from mice with or without Th9 adoptive transfer

Mouse endometriotic lesions were homogenized using ceramic beads (13113-50, Qiagen N.V., Hilden, Germany) in the Omni Bead Ruptor 24 (19-010, Omni International Inc). Total RNA was extracted and purified from lesion tissue lysate using Norgen Total RNA + Micro Isolation Kit (48500) as per manufacturer instructions. RNA concentrations were normalized to 120 ng/µL, with RNA concentration and purity verified using NanoDrop 2000 spectrometer (Thermo Fisher Scientific). Using the Mouse Fibrosis V2 Panel (NanoString, Seattle, WA, USA), 760 genes were evaluated in mouse lesion RNA. Hybridization, sample processing, and data collection were conducted by the Ontario Institute of Cancer Research (Toronto, ON, Canada). In brief, samples were hybridized with probes over an 18-h incubation, then loaded into the nCounter cartridge, and digital counts were obtained across 280 fields of view using the nCounter Digital Analyzer. Data were normalized to internal controls and housekeeping genes to ensure accurate quantification across samples using nSolver software. Any housekeeping genes with an average count <100 were removed and any genes with a maximum count <20 were not included in analysis. Evaluation of differentially expressed genes was conducted using nSolver software (version 4.0), in which heat maps were generated to display data in unsupervised clusters. Advanced analysis was conducted to detect patterns of differential expression in genes associated with specific functions, including Th subset differentiation and type 2 inflammatory responses. Through nSolver’s advanced analysis functions, Pathview analysis was conducted to illustrate significant changes to activity of specific pathways related to focal adhesion, chemokine signaling pathways, cytokine–cytokine receptor interaction, and PI3K-AKT signaling.

### Immunohistochemistry of mouse endometriotic lesions

Mouse endometriotic lesion tissues were fixed for 24 h in 4% paraformaldehyde at 4 °C before being transferred to 70% ethanol. Tissues were then dehydrated and paraffinized over 11 h and embedded in paraffin blocks. Sections were cut at 5 µm and mounted on glass slides for immunohistochemistry staining using a Leica Bond RX automated stainer (Leica Microsystems). Sections underwent citrate-based epitope retrieval for 20 min before 15 min incubation with primary antibodies for proliferation marker Ki67 (1:3,000; ab15580, Abcam) or angiogenic marker CD31 (1:300; 77699S, New England Biolabs). Leica BOND Polymer Refine Detection kit (Leica Microsystems) was used for DAB chromogen detection and hematoxylin counterstain. Slides were scanned at 40× magnification in an Olympus VS120 high-resolution slide scanner (Olympus Life Science). Images were analyzed with algorithms designed in HALO AI (Indica Labs, Albuquerque, NM, USA). Using the HALO tissue classifier feature, tissue was classified into slide glass (empty area), stroma, epithelium, vasculature, and glandular lumen. An area quantification algorithm was designed to analyze CD31 staining of vascular tissue areas, while a cytonuclear algorithm was designed to analyze Ki67^+^ cells. Percent positive cells or positive area from each tissue type were counted by their respective algorithms and data were imported to GraphPad Prism for statistical analysis. For CD68 immunohistochemical staining to evaluate macrophage presence, antigen retrieval was conducted for 30 min using citrate buffer and endogenous peroxidase activity was blocked. Sections were then stained for 30 min with an anti-mouse CD68 polyclonal Ab (1:500, ab125212, Abcam, UK) to detect macrophages/monocytes. Slides were digitally scanned for analysis as previously detailed^14^^,^^22^ prior to generating an area quantification algorithm in HALO AI (Indica Labs) to calculate percent positive stain for CD68.

### Immunofluorescence of mouse endometriotic lesions

Mouse endometriotic lesion tissues were sectioned at 5 µm and mounted on glass slides before undergoing deparaffinization and citrate-based heat antigen retrieval for 20 min. Slides were permeabilized in PBS-T for 10 min followed by peroxidase suppression for 10 min (35000, Thermo Fisher Scientific). After 1 h blocking in 5% bovine serum albumin PBS-T solution, slides were incubated with primary antibodies rabbit anti-mouse IL-9 (1:200; Ab203386, Abcam), rat anti-mouse CD4 (1:50; MA1146, Thermo Fisher Scientific), and chicken anti-GFP (1:100; A10262, Thermo Fisher Scientific) overnight at 4 °C. After three 10-min washes with PBS-T, slides were incubated with secondary antibodies donkey anti-rabbit Alexa Fluor 568 (1:400; A10042, Thermo Fisher Scientific), donkey anti-rat Alexa Fluor 647 (1:400; A78947, Thermo Fisher Scientific), and goat anti-chicken Alexa Fluor 488 (1:200; A11039, Thermo Fisher Scientific) for 2 h in the dark at room temperature. Finally, slides were washed thrice with PBS-T at 10 min per wash, then twice with PBS at 5 min per wash, before mounting with DAPI-containing SlowFade Glass mounting medium (S36920, Thermo Fisher Scientific). Slides were scanned using a Leica Mica confocal microscope and visualized using the LAS X Life Science Microscope Software (Leica Microsystems).

### Statistical analyses

For cytokine analyses, flow cytometric population comparison, and tissue microarray IL-9 stain comparison, one-way ANOVA tests were conducted in GraphPad Prism, version 10. For any comparison between only 2 groups (EMS vs sham, adoptive transfer vs PBS control), a Student t-test was conducted in GraphPad Prism. Significance was considered *P* ≤ 0.05. Outlier tests were conducted on each set of data (ROUT Q = 1%) and all statistical tests were performed on cleaned data.

## Results

### IL-9^+^ cells were significantly higher in lesions from patients with EMS compared to control endometrium

**Figure 1.**
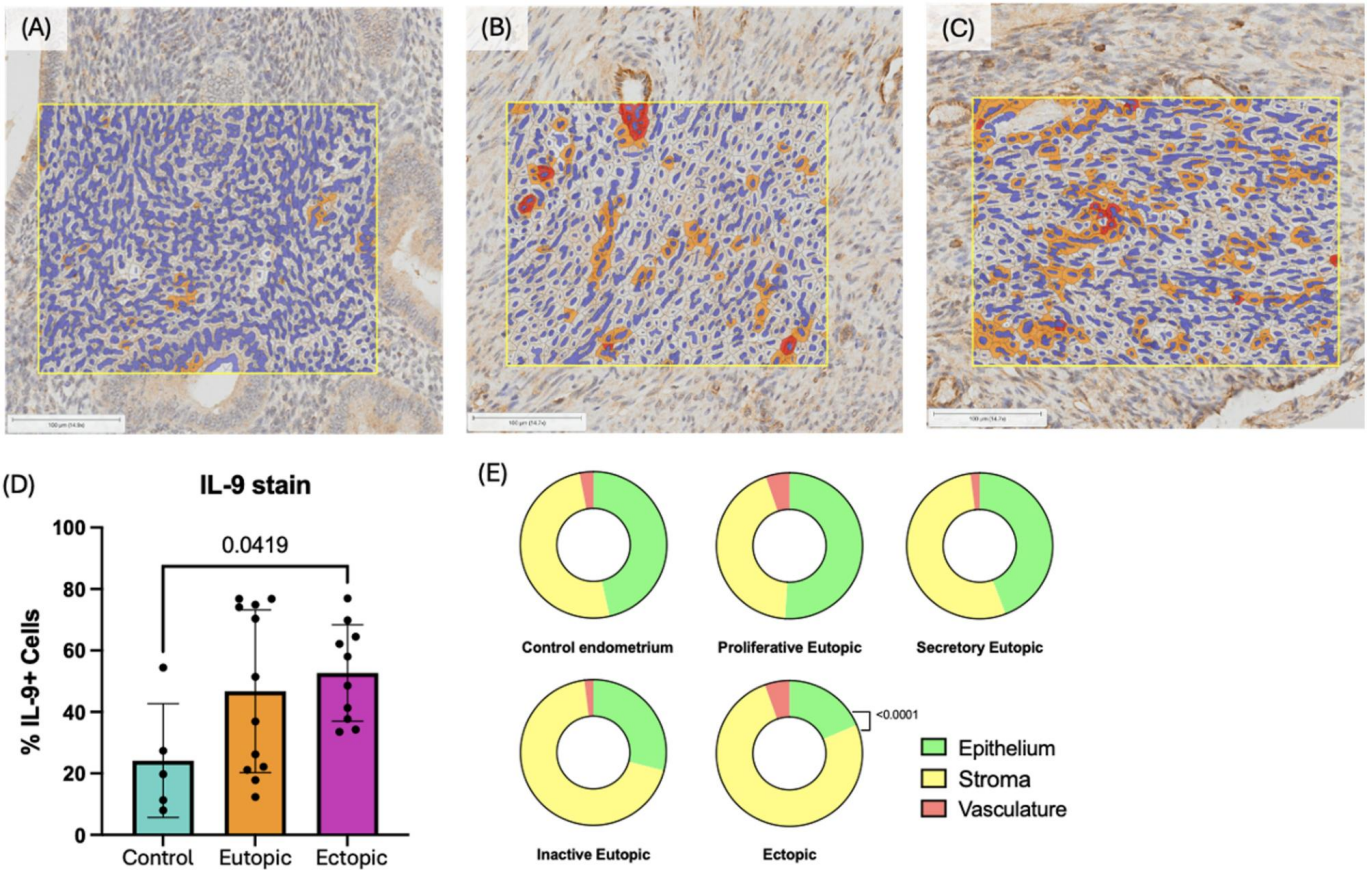
IL-9 presence in human endometrioma, eutopic endometrium, and control endometrium. Eighty-one cores in tissue microarray containing triplicates of control endometrium (*n* = 5), eutopic patient endometrium (*n* = 12), and endometrioma tissues (*n* = 10) were stained with anti-IL-9 antibody. Slides were scanned at 10× magnification; scale bars = 100 µm. HALO AI (Indica Labs) was used to classify tissue into classes of epithelium, stroma, and vasculature, with classes to exclude glandular lumen, glass, and debris from analysis. Cytonuclear analysis algorithm was optimized to detect weak (yellow), moderate (orange), and strong (red) anti-IL-9 stain. (A) Control healthy endometrium with cytonuclear analysis markup showing sparse staining for IL-9. (B) Eutopic endometrium from EMS patient with cytonuclear analysis markup showing positive IL-9 staining. (C) Endometrioma lesion with cytonuclear analysis markup showing positive IL-9 staining. (D) Percent cells positively stained for IL-9 in control endometrium, eutopic endometrium, and endometrioma lesion tissues. Staining of IL-9 in endometrioma tissues is significantly stronger than control endometrium (*P* = 0.0419). (E) Proportion of IL-9^+^ stromal cells was strongest in ectopic samples, significantly higher than epithelial proportion (*P* > 0.0001). This difference was not observed in control, proliferative eutopic, or secretory eutopic endometrium. Inactive endometrium (*n* = 2) showed a similar disparity in IL-9^+^ representation between stromal and epithelial cells. Statistical significance was determined by ordinary one-way ANOVA with Tukey multiple comparisons test.

To quantify IL-9 presence in EMS lesions, immunohistochemical staining for IL-9 was performed on a tissue microarray comprised of patient samples (ovarian EMS lesions, *n* = 10; eutopic endometrium, *n* = 12) and control endometrium samples (*n* = 5). All control endometrium samples were in proliferative phase. Among the 12 patient eutopic endometrium samples, 5 were in secretory phase, 5 were in proliferative phase, and 2 were inactive endometrium. Image analysis with optimized HALO AI cytonuclear algorithm found significantly higher percentage of IL-9^+^ cells (*P* = 0.0419) in EMS lesions (endometrioma) compared to control endometrium (Fig. 1D). Across proliferative and secretory eutopic patient endometrium and control endometrium samples, IL-9^+^ cells were found roughly to be evenly distributed between stroma and glandular epithelium, with a small percentage (0.2%–3%) represented within the endothelial cell compartments. In lesions, stromal cells represented the majority of IL-9^+^ cells, being significantly higher than IL-9^+^ epithelial cells (*P* < 0.0001) (Fig. 1E). While the *n* = 2 for inactive endometrium samples was insufficient to produce representative statistical results, the proportions of IL-9^+^-stained cells in inactive endometrium samples were most similar to results seen in lesion samples. Further, the proportion of IL-9^+^ cells represented by stromal cells in lesions was significantly higher than that of all other sample types, excluding inactive endometrium (Fig. S1).

### Changes in cytokine secretion profiles of human CD4^+^ lymphocytes given Th9-driving growth factors

#### IL-7 increased secretion of proinflammatory, chemotactic, and angiogenic cytokines by Th9-driven CD4^+^ human lymphocytes

**Figure 2.**
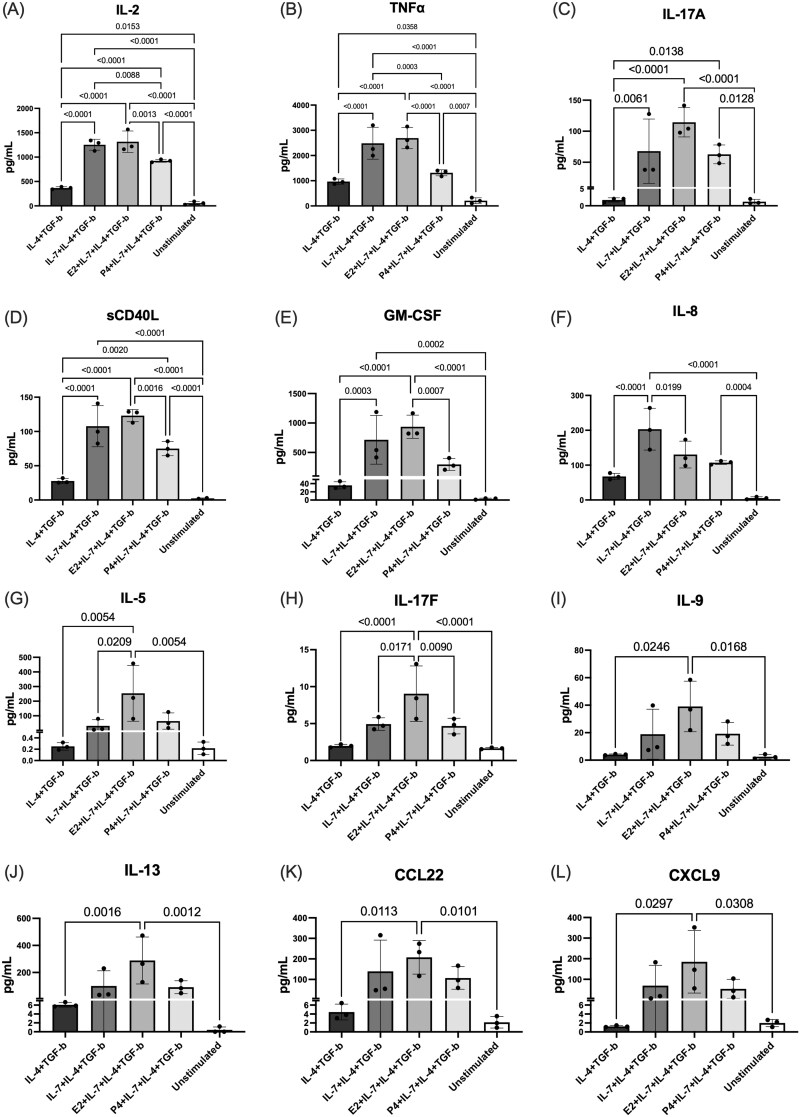
Cytokine response by PBMC-derived Th9-driven T cells to different growth factor cocktails. CD4^+^ T cells were negatively selected from human PBMCs by immunomagnetic separation and cultured under Th9-polarizing conditions (IL-2, IL-4, TGF-β, anti-CD3ε, anti-CD28, anti-IFNγ) in the presence or absence of IL-7 and/or hormonal treatment (E2; 17-β-estradiol [1 × 10^−6^ M], P4; progesterone [1 × 10^−6^ M]), alongside unstimulated controls (no growth factor cocktail). Cells were restimulated with PMA and ionomycin for 5 h prior to supernatant collection. (A–L) Cytokine concentrations were quantified using a 32-plex multiplex cytokine array (Eve Technologies). Statistical significance was determined by ordinary one-way ANOVA with Tukey multiple comparisons test.

Using human PBMCs isolated from healthy volunteer blood, CD4^+^ T cells were isolated by immunomagnetic negative selection and cultured with Th9 growth factors (IL-4 and TGF-β) to drive them toward a Th9 phenotype. With the aim of optimizing Th9 cell differentiation, IL-7 was included in the Th9-driving cocktail based on literature suggesting that IL-7 increases IL-9 production by Th9 differentiated cells in vitro.^19^ Indeed, groups that received IL-7 had significantly increased concentration of IL-2, IL-8, IL-17A, sCD40L, GM-CSF, and TNF-α (Fig. 2A–F).

#### Estrogen and progesterone modulate the secretory profile of Th9-driven CD4^+^ human lymphocytes

To evaluate the impact of E2 and P4 hormones on the secretory profile of human PBMC–derived Th9-driven T cells, groups were treated with Th9 growth factors (IL-7, IL-4, TGF-β), in combination with either 1.0 × 10^−6^ M E2 or 1.0 × 10^−6^ M P4 for 24 h. Multiplex cytokine analysis of cell culture supernatant revealed that the addition of P4 significantly decreased IL-2 and TNF-α levels (Fig. 2A, B), while E2 significantly increased levels of IL-5 and IL-17F and decreased IL-8 (Fig. 2F–H) compared to cells treated with the Th9-driving cocktail but not E2 or P4. These findings suggest that estrogen (E2), and to a lesser extent progesterone (P4), modulate the secretory profile of in vitro–derived human Th9 cells.

### Evaluation of baseline Th9 presence and immune cell IL-9R positivity in mice induced with endometriosis vs sham-operated controls

In initial investigations to capture baseline Th9 cell presence in our syngeneic mouse model of EMS, we used flow cytometry to evaluate splenic and peritoneal Th9 cell numbers. Our Th9 panel captured negligible Th9 cell presence in the peritoneal and splenic immune cell populations (data not shown). This was not unexpected, as Th9 cells differentiate in the periphery in response to stimuli, and these mice had only received surgical induction of EMS (or sham) without additional stimulation that would drive Th9 differentiation. In a separate flow cytometry panel, we quantified IL-9 receptor (IL-9R) expression on peritoneal immune cells, including MCs and MCcps, to gauge baseline differences in EMS. Significant results from this panel are elaborated below.

#### IL-9R^+^ peritoneal immune cells were increased in endometriosis-induced mice compared to sham-operated controls

**Figure 3.**
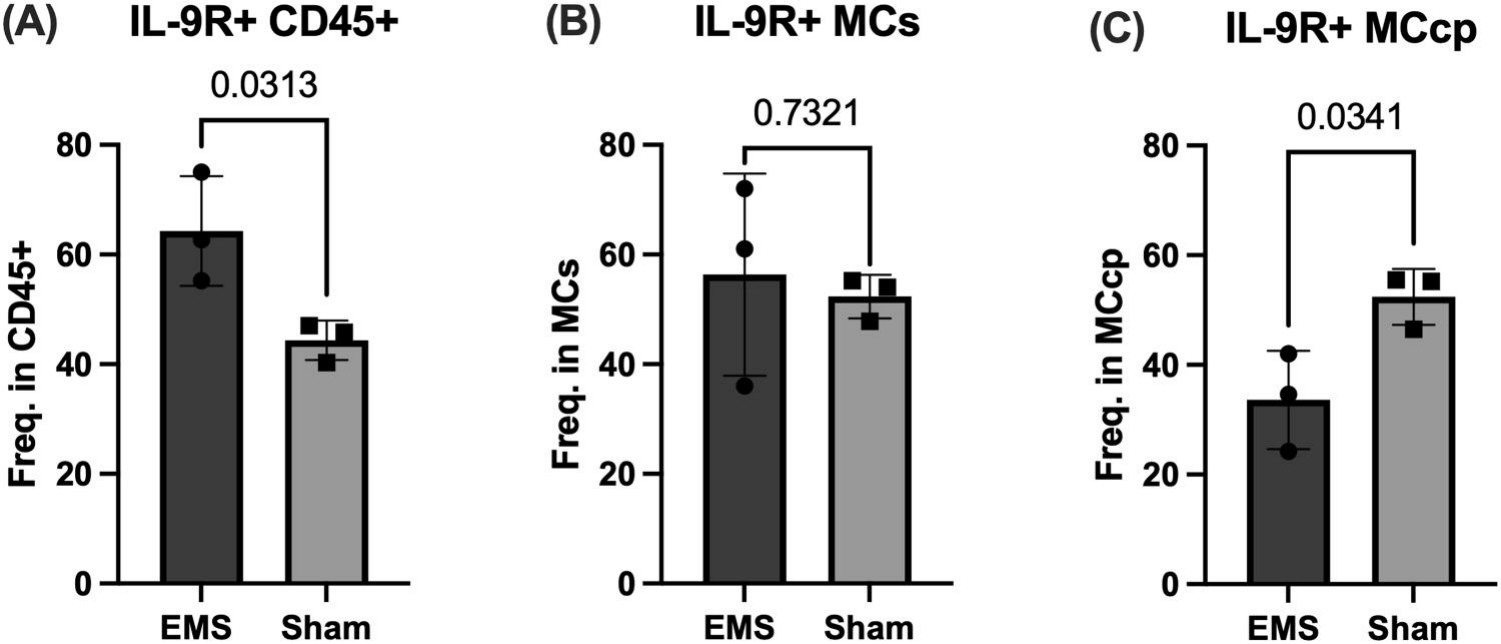
IL-9R^+^ peritoneal immune cell populations of mice induced with endometriosis or sham-operated controls. Peritoneal cells collected from mice induced with EMS (or sham-operated controls) were evaluated by flow cytometry for markers of MCs, MCcps, and IL-9R. (A) IL-9R^+^ cells were significantly higher in CD45^+^ peritoneal cells of mice induced with EMS compared to sham-operated controls. (B) No significant difference in IL-9R expression within MCs in EMS-induced mice vs sham-operated controls. (C) Of the MCcp populations in each group, a significantly higher portion were IL-9R^+^ in sham-operated mice compared to EMS-induced mice. Statistical significance was evaluated with unpaired t-tests.

Flow cytometric analysis was used to evaluate the presence of IL-9R expression in peritoneal immune cells of mice induced with EMS vs sham-operated controls. Compared to sham mice, EMS-induced mice showed significantly higher presence of IL-9R^+^ cells within peritoneal CD45^+^ cells (*P* = 0.031, Fig. 3A). As multiple pathogenic mechanisms of Th9 cells have been reported to depend upon IL-9 stimulation of MCs,^23^^,^^24^ IL-9R expression was evaluated in both MCs and MCcp. No significant differences were observed in peritoneal or splenic MC or Th9 cell frequency when comparing EMS and sham mice (data not shown). Moreover, no difference in MC IL-9R expression was found between groups (Fig. 3B), suggesting that disease status does not alter IL-9 responsiveness of differentiated MCs.

Within MCcp populations (immature MC precursors), IL-9R expression was significantly lower in EMS-induced mice compared to sham-operated mice (*P* = 0.034, Fig. 3C). While the overall MCcp population was significantly larger within peritoneal immune cells of sham controls compared to EMS mice (*P* = 0.00441, data not shown), this stark difference in IL-9R expression is noteworthy considering that IL-9R expression in peritoneal CD45^+^ cells was higher in EMS mice. Ultimately, these results suggest that while IL-9 signaling may contribute to peritoneal inflammation through mature MCs, EMS may disrupt MCcp homeostasis/differentiation, potentially reflecting altered recruitment, survival, and/or maturation of MCcps within the inflammatory milieu. This supports a role for dysregulated IL-9–mediated immune interactions in EMS, rather than a uniform upregulation of IL-9 responsiveness across MC lineages. In addition, another panel was used to identify Th9 cells; however, splenic and peritoneal Th9 cell numbers were negligible in both groups. As Th9 cells generally differentiate in the periphery,^5^^,^^25^ this may account for these low cell counts.

### Changes caused by adoptive transfer of Th9-like cells into mice induced with endometriosis

In our adoptive transfer experiment, mice were induced with EMS (day 0), and on day 7 mice received an intraperitoneal injection of either PBS or ∼274,000 GFP^+^, in vitro*–*expanded, purified Th9-like lymphocytes suspended in PBS. Results of this experiment, as detailed below, captured changes to the inflammatory profile and lesion landscape in mice that received the adoptive transfer as compared to mice that received PBS.

#### In vitro derivation of Th9 cells from mouse splenocytes

**Figure 4.**
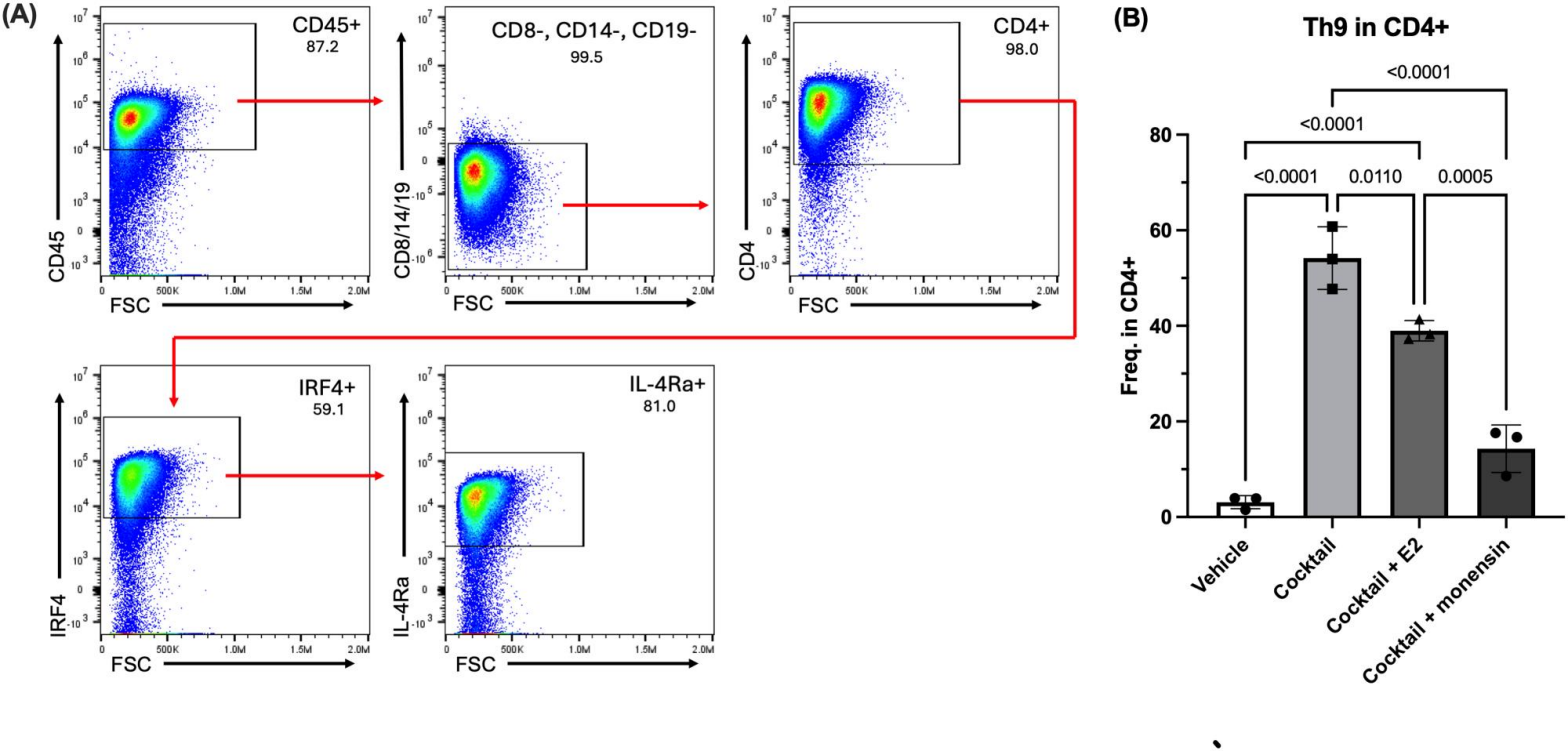
Deriving Th9 phenotype in vitro from mouse splenocytes. CD4^+^ cells were negatively selected using immunomagnetic separation and cultured with a cocktail of growth factors (IL-2, IL-4, TGF-β, anti-CD3, anti-CD28, anti-IFNγ). Cells were stimulated with PMA and ionomycin for 6 h. (A) Gating strategy for selecting Th9 phenotype. Out of the CD45^+^ population, cells negative for CD8α, CD14, and CD19 were selected. The CD4^+^IRF4^+^IL-4Rα^+^ cells within this CD45^+^CD8a^−^CD14^−^CD19^−^ population were considered Th9 cells. (B) One-way ANOVA with Tukey multiple comparisons test was used to evaluate statistical significance of Th9 phenotype yield in different culture conditions.

To gain insights into the influence of adoptively transferred Th9 cells on the immune microenvironment of mouse EMS lesions, we first aimed to derive Th9 cells in vitro from mouse splenocytes. Using an optimized format of Pham’s protocol^18^ to derive Th9 cells, the Th9 growth cocktail-treated group yielded significantly higher Th9 cells compared to those without Th9 growth factors as well as the monensin-treated group (*P* ≤ 0.05, Fig. 4B). Monensin is a protein transport inhibitor used to prevent secretion of soluble cytokines and allow for intracellular staining, and its reduction of Th9 cell yield likely occurred because Th9 cells require autocrine IL-2 and TGF-β stimulation for phenotype development.^18^ Treating the Th9-driven group with E2 (1 × 10^−6^) had no significant impact on Th9 yield, but showed a slight downward trend compared to the group treated only with Th9 growth factors. The confirmation of mouse Th9 cell fate and expansion in vitro carried forward subsequent adoptive transfer experiments in our mouse model of EMS. Cells that were CD8α^−^/CD14^−^/CD19^−^/CD4^+^/IRF4^+^/IL-4Rα^+^ were considered Th9 cells (Fig. 4A).

#### IL-1α significantly increased in plasma following Th9 adoptive transfer in a mouse model of endometriosis

**Figure 5.**
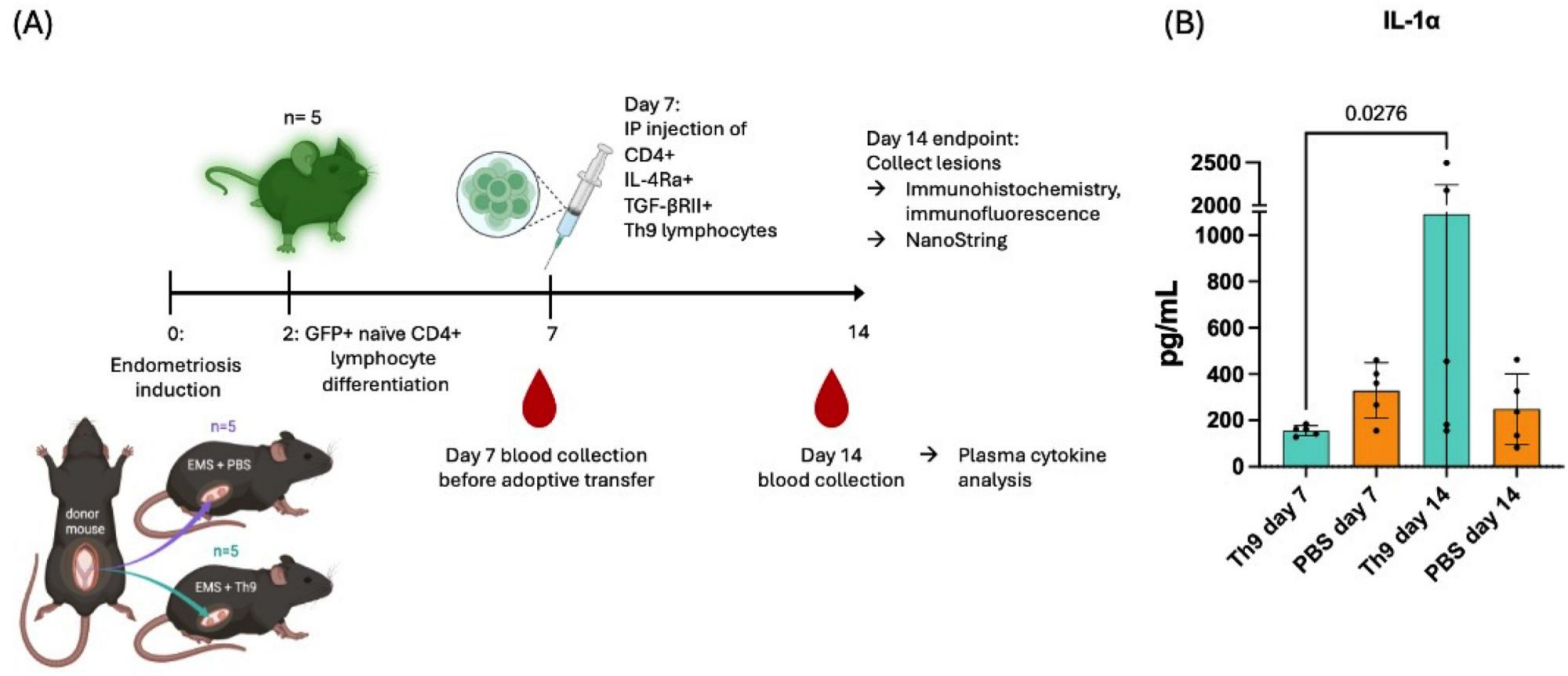
Endometriosis mouse model with adoptive transfer of GFP^+^ Th9 cells or PBS. (A) Workflow of EMS mouse model with adoptive transfer of Th9-like cells. Mice were induced with EMS on day 0. GFP^+^ mouse splenic CD4^+^ T cells were cultured in vitro with Th9 phenotype-deriving growth factors and double positively selected for IL-4Rα and TGF-βRII by immunomagnetic separation. On day 7, mice were injected intraperitoneally with suspensions of 2.74 × 10^5^ Th9-like lymphocytes or PBS. (B) Mouse plasma collected at 7- and 14-day timepoints underwent multiplex analysis for inflammatory cytokine levels. Statistical significance was determined using one-way ANOVA with Tukey multiple comparisons test. IL-1α significantly increased in the Th9 adoptive transfer group from day 7 to day 14 while no change was observed in the PBS control group. “Th9” is the mouse group that received adoptive transfer of Th9-like lymphocytes; “PBS” is the PBS control mouse group.

To investigate the systemic immune response associated with adoptive transfer of Th9-like cells in a mouse model of EMS, plasma cytokine levels were measured at key time points following disease induction. Blood collection on days 7 and 14 enabled comparison of cytokine dynamics before and after Th9-like cell transfer. Analysis revealed that IL-1α was significantly increased (*P* = 0.0276) between day 7 and day 14 in the Th9-like adoptive transfer group, while no changes were observed between these time points for the PBS control group (Fig. 5B). This suggests that adoptively transferred Th9 cells were likely exerting a proinflammatory response captured in the systemic circulation.

#### Th9 cells infiltrated endometriotic lesions upon adoptive transfer in a mouse model of endometriosis

**Figure 6.**
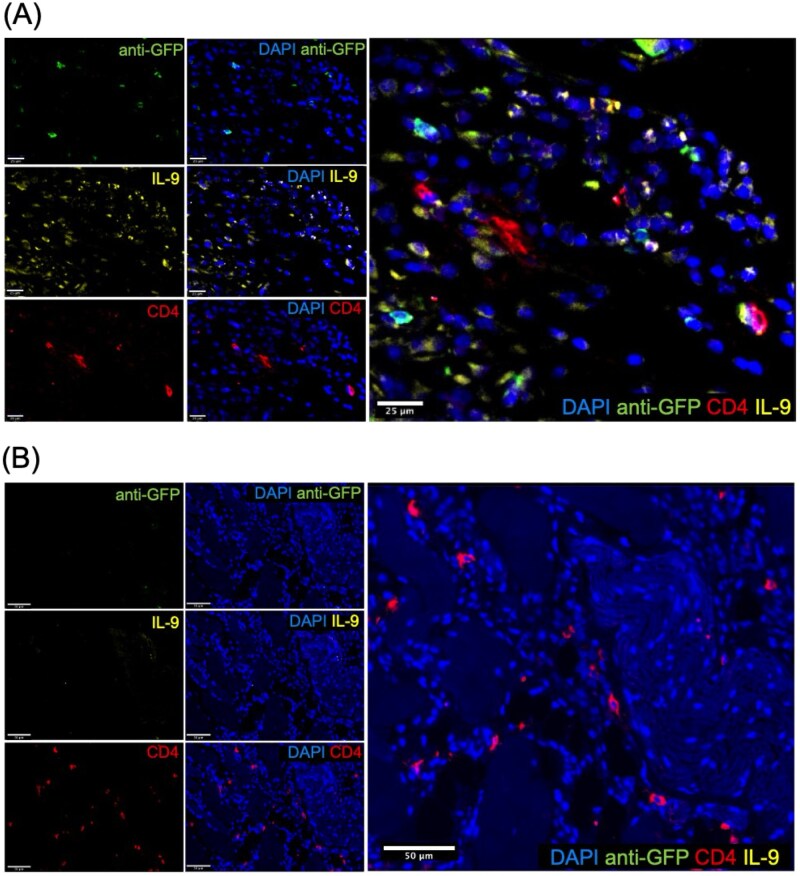
Adoptively transferred GFP^+^ Th9-like cells infiltrated into mouse endometriosis lesion tissue. (A) GFP^+^CD4^+^IL-9^+^ cells observed in lesions of mice that received Th9 cells. Scale bar = 25 µm. (B) PBS control mouse lesion displayed some CD4^+^ and IL-9^+^ staining but lacked GFP^+^ signal. Scale bar = 50 µm. Blue, DAPI nuclear stain; green, anti-GFP–Alexa Fluor 488; yellow, anti-IL-9–Alexa Fluor 568; red, anti-CD4–Alexa Fluor 647.

Following adoptive transfer of GFP^+^ Th9-like lymphocytes into mice induced with EMS, we aimed to establish whether these adoptively transferred cells infiltrated into endometriotic lesions. Indeed, we identified GPF^+^CD4^+^IL-9^+^ cells incorporated into endometriotic lesions through fluorescent microscopy of mouse lesion tissue (Fig. 6). GFP^+^ cells were evenly distributed throughout stromal compartments, with very few cells localized to epithelial compartments. This qualitative observation supports the notion that adoptively transferred Th9-like cells likely contribute to lesion pathology and associated microenvironmental changes.

#### Mouse endometriotic lesion proliferation was significantly impeded following Th9 adoptive transfer

**Figure 7.**
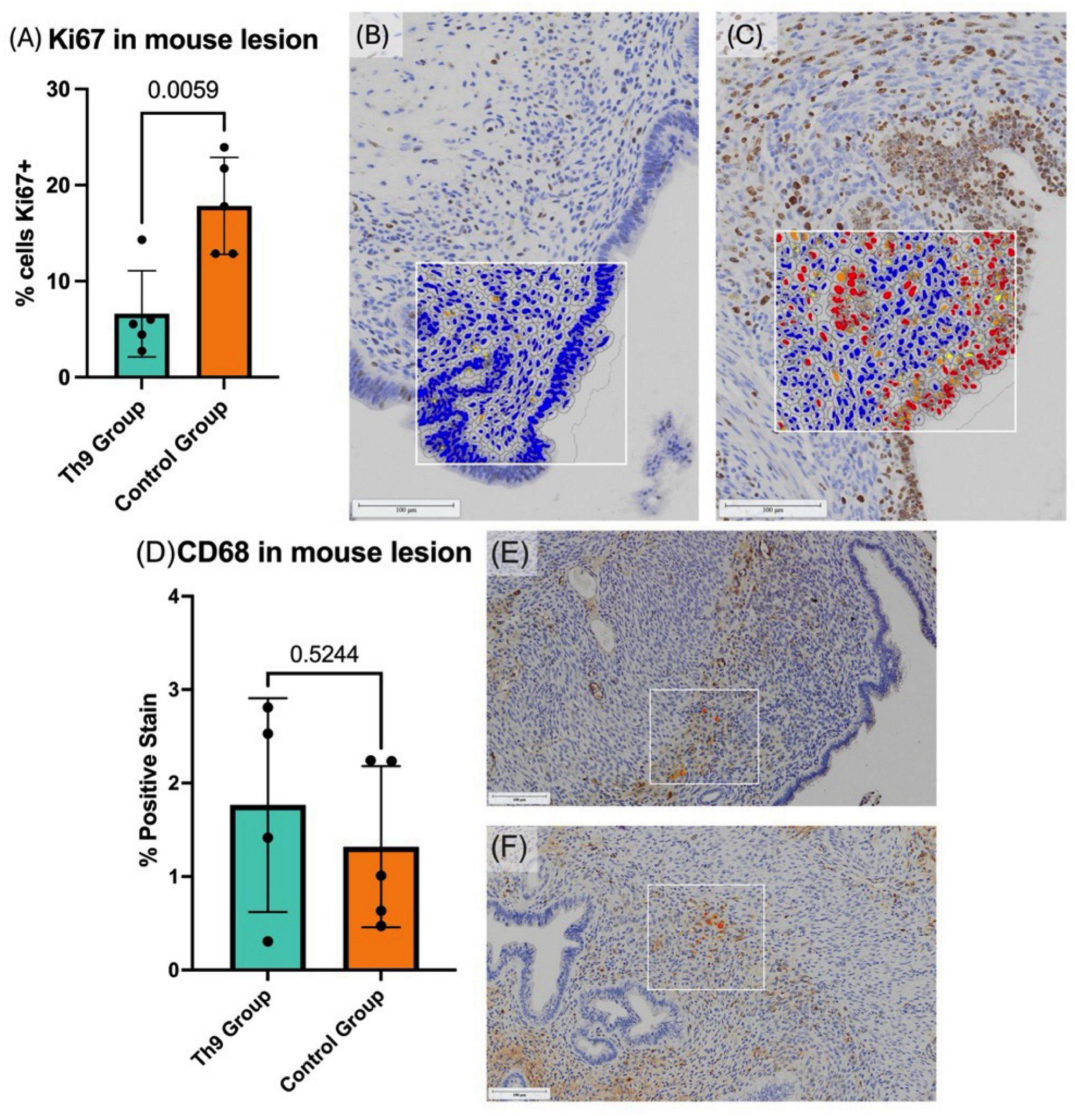
Ki67 and CD68 immunohistochemical staining in endometriotic lesions of mice that received adoptive transfer of Th9-like cells or PBS control. (A) Percentage of proliferative (Ki67^+^) cells was significantly higher in the PBS control group (*n* = 5) compared to the Th9 group (*n* = 5). (B) Ki67 staining of endometriotic lesion tissue in a mouse that received adoptive transfer of Th9-like cells. (C) Endometriotic lesion tissue in a mouse that received PBS control injection. Ki67 staining in (C) reflects a proliferative profile typical of EMS lesions in this model. (D) Area quantification of percentage of positive stain for CD68 was calculated for lesions from Th9 adoptive transfer (*n* = 4) and control (PBS; *n* = 5) groups. There was no significant difference in CD68^+^ percent-stained area between groups. (E) CD68 staining of endometriotic lesion tissue from a mouse that received adoptive transfer of Th9-like cells. (F) CD68 staining of endometriotic lesion tissue from a control mouse that received PBS. Statistical significance was assessed using an unpaired t-test. Scanned immunohistochemical images were digitally analyzed using HALO imaging software (Indica Labs) and provided at 10× magnification. Scale bars = 100 µm.

Proliferation is a key feature of endometriotic lesion establishment and progression, contributing to tissue expansion and persistence. Given that Th9 cells have been implicated in modulating cell proliferation in various biological contexts, assessing their impact on proliferative activity within endometriotic lesions is critical. Therefore, immunohistochemical staining for Ki67, a well-established marker of cellular proliferation, was performed on mouse lesion tissues to evaluate whether adoptively transferred Th9-like cells influence proliferative dynamics within the lesion microenvironment. Analysis revealed significant reduction (*P* = 0.0059) of Ki67 immunostaining (mean percentage of Ki67^+^ cells) in the lesions of mice that received adoptive transfer of Th9-like lymphocytes as compared to the PBS control group (Fig. 7A–C). Since T helper cells are often associated with macrophage activation,^26^ we performed immunohistochemistry on lesion tissues to stain for macrophage marker CD68. There were no significant changes in CD68^+^ stain in lesions from mice in the adoptive transfer group and the PBS control group (Fig. 7D).

### Adoptive transfer of Th9 cells altered the transcriptional profile of mouse endometriotic lesions

**Figure 8.**
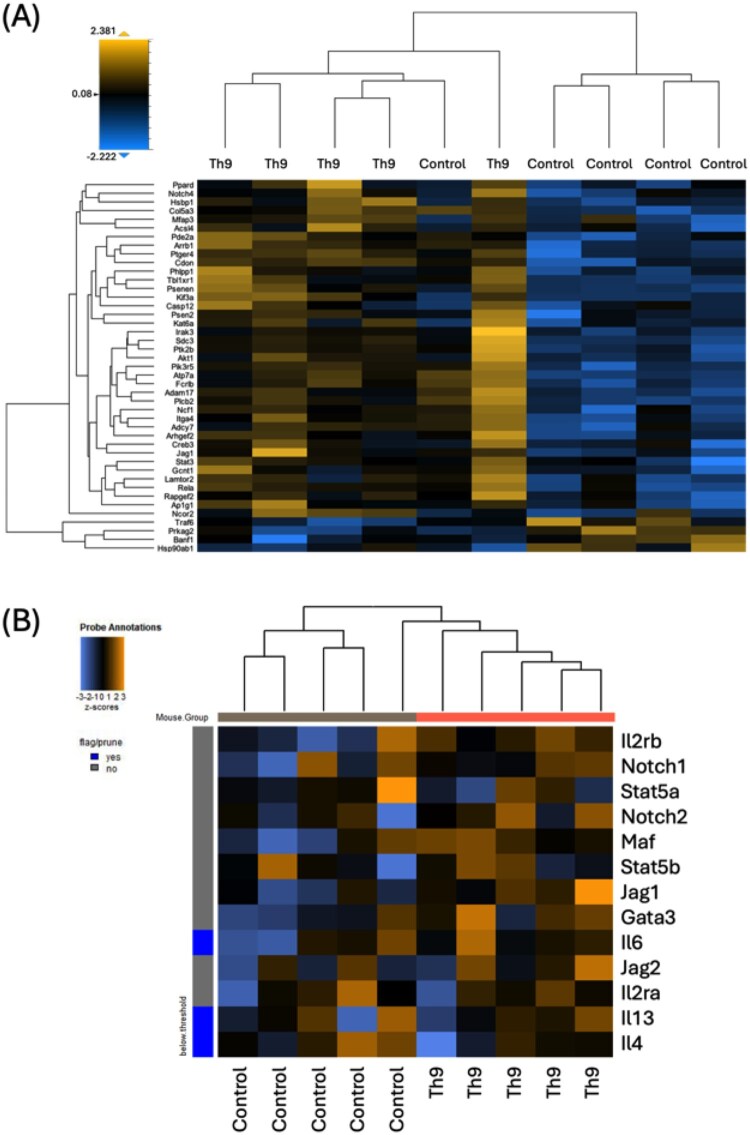
Heat map of significant differentially expressed genes in mouse endometriotic lesions of mice that received Th9 adoptive transfer or PBS. RNA samples isolated from endometriotic lesion tissues of mice that received adoptive transfer of Th9-like cells (*n* = 5) or PBS (*n* = 5) were evaluated for expression of 760 genes using NanoString’s nCounter Mouse Fibrosis V2 panel. Samples were analyzed with unsupervised clustering. Dendogram across the top of the heat map shows clustering of samples almost exclusively into separate groups. (A) Thirty-nine genes of the panel were significantly upregulated in the Th9 adoptive transfer group while 4 were significantly downregulated. (B) Genes involved in Th2 differentiation were generally upregulated in the Th9 adoptive transfer group compared to the control group. *Il4*, *Il6*, and *Il14* expression fell below the threshold to be distinguished from background signal as indicated by blue vs gray flag on the left border of the heat map. Using unsupervised clustering, samples clustered together with their respective treatment groups. Orange indicates increased expression; blue, decreased expression.

To elucidate the functional impact of adoptively transferred Th9-like cells within the mouse EMS lesion microenvironment, it is critical to evaluate the molecular changes they induce at the site of inflammation or pathology. Total RNA was extracted from lesion tissues and analyzed using the NanoString nCounter Mouse Fibrosis V2 panel, which enables comprehensive profiling of gene expression related to fibrosis, inflammation, extracellular matrix (ECM) remodeling, and immune responses. This panel includes a broad list of genes involved in inflammation, immunity, and fibrosis. Analysis of data in nSolver software revealed that 43 genes were significantly differentially expressed (*P* ≤ 0.05). Among them, 39 genes were significantly upregulated in the Th9 adoptive transfer group, and 4 genes were significantly downregulated (*Banf1*, *Hsp90ab1*, *Prkag2*, *Traf6*; respective fold changes: 0.803, 0.794, 0.777, 0.855) (Fig. 8A). Samples were analyzed with unsupervised clustering, but clustered almost exclusively within their respective treatment groups because of their similarities in gene expression profiles. Of the 39 genes significantly upregulated in lesions of the Th9 adoptive transfer group, 20 genes were associated with the process of proliferation and 15 were associated with inflammation. Several pathways and themes are represented in these results. Namely, 3 genes associated with ECM formation (*Col5a3*, *Itga4*, and *Mfap3*; respective fold changes: 1.129, 1,093, 1,030), 6 genes relevant in Notch signaling (*Adam17*, *Jag1*, *Notch4*, *Ppard*, *Psenen*, *Psen2*; respective fold changes: 1.080, 1.141, 1.132, 1.060, 1.032, 1.006), and 7 genes associated with the adenosine pathway (*Adcy7*, *Arhgef2*, *Arrb1*, *Pde2a*, *Plcb2*, *Ptger4*, *Sdc3*; respective fold changes: 1.105, 1.050, 1.103, 1.077, 1.296, 1.201, 1.445) were significantly upregulated in the lesions of the Th9 adoptive transfer group. Five genes involved in PI3K-Akt signaling (*Pi3kr5*, *Akt1*, *Phlpp1*, *Itga4*, *Creb3*; respective fold changes: 1.527, 1.051, 1.111, 1.093, 1.036) were significantly upregulated while another PI3K-Akt–associated gene, *Hsp90ab1* (fold change: 0.794), was downregulated in the Th9 cell adoptive transfer group. As Th cells are commonly known to activate macrophages, we also conducted additional analysis to gain insights into M1 and M2 polarization status (Fig. S2). However, no clear unsupervised clustering of treatment groups was observed.

#### Th2 differentiation transcripts were altered in lesions of mice who received Th9 cell adoptive transfer

Advanced analysis in nSolver software was conducted to identify consistent trends in gene expression. Genes associated with Th2 differentiation (*Gata3*, *Il13*, *Il2ra*, *Il2rb*, *Il4*, *Il6*, *Jag1*, *Jag2*, *Maf*, *Notch1*, *Notch2*, *Stat5a*, *Stat5b*) were found to be upregulated in the Th9 adoptive transfer group, with *Jag1* significantly increased (Fig. 8B). *Il4*, *Il6*, and *Il14* expression fell below the threshold to be distinguished from background signal as indicated by blue vs gray flag on the left border of the heat map. *Stat3*, though not included in this heat map, is required for Th2 development and was significantly upregulated in the Th9 adoptive transfer group. This preliminary analysis further supports the influence of Th9 cells on the Th2 inflammatory response.

#### Gene pathways for focal adhesion, PI3K-Akt, chemokine signaling, and cytokine–cytokine receptor interaction were altered in endometriotic lesions of Th9 cell adoptive transfer group

Within the nSolver advanced analysis, Pathview analysis highlighted a significant difference in genes involved in focal adhesion, wherein the expression levels of several genes were lower in the PBS control group as compared to the Th9 adoptive transfer group—specifically, genes within the integrin α (ITGA) family, receptor tyrosine kinase, with downstream effectors including Src, Crk, and Rac.

## Discussion

Our group has extensively reported on various pathways driving immune dysregulation in EMS, including the involvement of estrogen-mediated MCs and the dysregulated IL-23/Th17 axis.^14^^,^^15^ Given the well-documented Th2 immune bias in EMS^2^^,^^27^^,^^28^ and the role of Th9 cells in regulating Th2 responses via IL-9 secretion, we aimed to investigate, for the first time, the role of Th9 cells in EMS pathophysiology, particularly due to their established cooperation with MCs and other immune cells to perpetuate inflammatory diseases.^8^^,^^24^ Notably, IL-9 supports MC development and function, and emerging evidence suggests interactions between IL-9 and IL-33 signaling in type 2 inflammation. Indeed, Blom et al. identified that human CD4^+^ T cells can be induced by IL-33 to secrete IL-9.^28^^,^^29^ Moreover, Du et al. identified IL-9 to be a downstream effector of IL-33 in airway inflammation, with IL-9–deficient mice depicting significantly attenuated pathological changes following IL-33 stimulation.^30^ Previously, we have established the role of IL-33 in activating group 2 innate lymphoid cells in EMS progression.^31^ However, the inflammatory relationship between IL-9 and IL-33 has yet to be explored in EMS pathophysiology.

In this study, we observed increased IL-9 protein within human endometriotic lesions, consistent with literature depicting increased IL-9 in EMS patient PF and plasma.^11^^,^^12^ Stromal cells comprised a significantly increased proportion of IL-9^+^ cells within lesions, illustrating a distinction in IL-9 distribution as compared to matched eutopic and control endometrium. Given that inactive endometrium and advanced lesions are characterized by increased fibrosis,^32^ the potential fibrotic influence of IL-9 in EMS should be investigated further. While our finding of IL-9 presence is significant, it is important to note that only ovarian (endometrioma) lesions were utilized. Due to the well-known heterogeneity and hormonal-dependent nature of EMS, there remains a need to investigate the presence and involvement of IL-9 in all phenotypes and stages of EMS, with samples stratified by menstrual stage. Moreover, it is important to recognize ubiquitous limitations in staging, with the revised American Society of Reproductive Medicine staging system most widely accepted.^33^ As literature shows that EMS stage poorly correlates with symptomology,^34^ results stratified by EMS stage may still not correlate with observed severity/clinical presentation, further complicating interpretations of results.

Previously, we showed that Ishikawa cells (endometrial adenocarcinoma cell line analog of endometrial stromal cells) treated with IL-17A significantly increased IL-9 production.^35^ This is notable as IL-17A is known to be increased and associated with EMS.^36^^,^^37^ Further providing a basis for this work, we have also depicted increased IL-9 levels in EMS patient plasma compared to controls.^12^ Thus, we aimed to gain insights into the complex biology of Th9 cells. First, human PBMC-derived CD4^+^ T cells were driven to a Th9 phenotype using a Th9 growth factor cocktail, including IL-7, IL-4, and TGF-β. As others have demonstrated the importance of estrogen and estrogen receptor α in T-cell functioning and activation,^38^^,^^39^ cells were further treated with E2 or P4. Cytokine profiling revealed that IL-7 stimulation specifically increased levels of IL-2, TNF-α, IL-17A, sCD40L, GM-CSF, and IL-8, supporting literature that IL-7 regulates T-cell development and homeostasis.^40^ Further stimulation with E2 significantly decreased IL-8 and increased IL-5 and IL-17F levels, whereas P4 significantly decreased IL-2 and TNF-α. This profile suggests that estrogen modulates Th9-associated effector programs, promoting Th2- and Th17-associated profiles. Given the estrogen-dependent nature of EMS, these data suggest a role of estrogen, as well as progesterone, in regulating Th responses/functional plasticity. While these results were observed in PBMC-derived Th9 cells, directly reflecting systemic immune programming, this may also provide insight as to how hormonal cues may influence Th9-associated responses within the local immune microenvironment. However, it is important to highlight the complex interactions between various immune cells, cytokines, and growth factors in shaping the chronic nature of EMS-associated inflammation, as discussed in several of our previous review articles.^2^^,^^3^^,^^41^ Further single-cell analyses are required to directly compare systemic and peritoneal Th9 cell populations in EMS.

As several of the documented pathogenic roles of Th9 cells are exerted through their support of MCs with IL-9,^23^^,^^42^ we analyzed IL-9R expression in MCs from the PF of EMS-induced mice. While IL-9R expression on MCs was unchanged, IL-9R expression within peritoneal CD45^+^ cells was significantly increased in EMS mice compared to sham controls. This is relevant to findings from Tarumi et al., who reported that IL-9R immunostaining was significantly higher in ovarian endometriotic lesions compared to eutopic and control endometrium.^13^ It is necessary to further examine which cell types comprise this IL-9R^+^ population to understand how IL-9 signaling impacts the immune microenvironment of EMS.

Adoptive transfer of GFP^+^ Th9 cells into EMS-induced mice resulted in migration of GFP^+^CD4^+^IL-9^+^ cells into lesions as well as reduced proliferation in lesions. These antiproliferative effects were depicted in both immunohistochemical staining (Ki67) of mouse lesions and within the analysis of differently expressed genes in RNA from mouse lesions. Within lesions of the Th9 adoptive transfer group, 20 of 39 of the significantly upregulated genes were associated with proliferation, whereas 15 genes were associated with inflammation. While few Th2-associated genes were significantly differentially expressed, advanced nSolver analysis revealed an overall enrichment of this transcriptional profile in the Th9 adoptive transfer group. This is consistent with known plasticity between Th9 and Th2 cells, as well as our finding of significantly increased expression of *Stat3*, a critical driving factor in Th2 differentiation that is also activated in Th9 differentiation.^5^

Six genes relevant in Notch signaling (*Adam17*, *Jag1*, *Notch4*, *Ppard*, *Psenen*, *Psen2*) were significantly increased in the Th9 adoptive transfer group, supporting prior studies implicating Notch signaling in Th9 development and EMS progression.^43–45^ Additionally, 3 genes associated with ECM formation, *Col5a3*, *Itga4*, and *Mfap3*, were also significantly increased in the Th9 adoptive transfer group. Fibrosis is characterized by excess accumulation of ECM components, supporting a potential role for Th9 cells in fibrotic remodeling in EMS, consistent with findings from other inflammatory diseases.^46^

Finally, several results also suggest a correlation between Th9 cells and activated IL-1 pathway signaling in EMS, as plasma IL-1α was significantly increased following Th9 adoptive transfer in mice and *Irak3*, the gene for IL-1 receptor–associated kinase 3, was upregulated in EMS lesions from the Th9 adoptive transfer group. Moreover, using Pathview analysis in nSolver, we found multiple components of the IL-1 signaling pathway to be increased in the Th9 adoptive transfer group; IL1B (IL-1β), IL1R1 (IL-1 receptor type 1), and IL1RAP (IL-1 receptor accessory protein). Notably, IL-1β stimulation is a pivotal determinant of exhaustion resistance in Th9 populations in melanoma tumors and has also been well documented for its elevated presence in EMS lesions.^47^^,^^48^ In humans, IL-1α is also significantly increased in patient PF and associated with advanced disease, while its receptor antagonist, IL-1Ra, is increased during earlier stages of EMS^49^ and can be produced by endometrial epithelial cells.^50^^,^^51^ Collectively, the coexistence of reduced lesion proliferation with increased inflammatory and fibrotic transcriptional programs in the Th9 adoptive transfer group is consistent with hallmark features of mature/advanced EMS lesions, characterized by diminished proliferative capacity, chronic inflammation, and progressive fibrotic remodeling. In this context, the prominence of IL-1 signaling and fibrosis in advanced EMS suggests that Th9 cells may exert distinct local and systemic effects dependent on disease stage and phenotype.

In conclusion, for the first time in literature, this study identifies Th9 cells as contributors to EMS-associated immune dysregulation. Collectively, results suggest that Th9 cells exhibit diverse, context-dependent roles, possibly facilitating MC-driven inflammation in early disease, while contributing antiproliferative functions in later stages of EMS. However, this study did not examine direct mechanistic evidence linking Th9-derived IL-9 and MC activation, which remain an important area for future investigation.

## Supplementary Material

vkag123_Supplementary_Data

## Data Availability

Data sets are available from the authors upon request.
