# Supplementary material for: Contributions of T-helper 9 cells in endometriosis-associated inflammation and lesion growth
Source: J Immunol. 2026 May 29;215(5):vkag123. doi: 10.1093/jimmun/vkag123 (PMC13220158; doi:10.1093/jimmun/vkag123)
Supplement: vkag123_Supplementary_Data [file vkag123_supplementary_data.pdf]

Supplemental figures

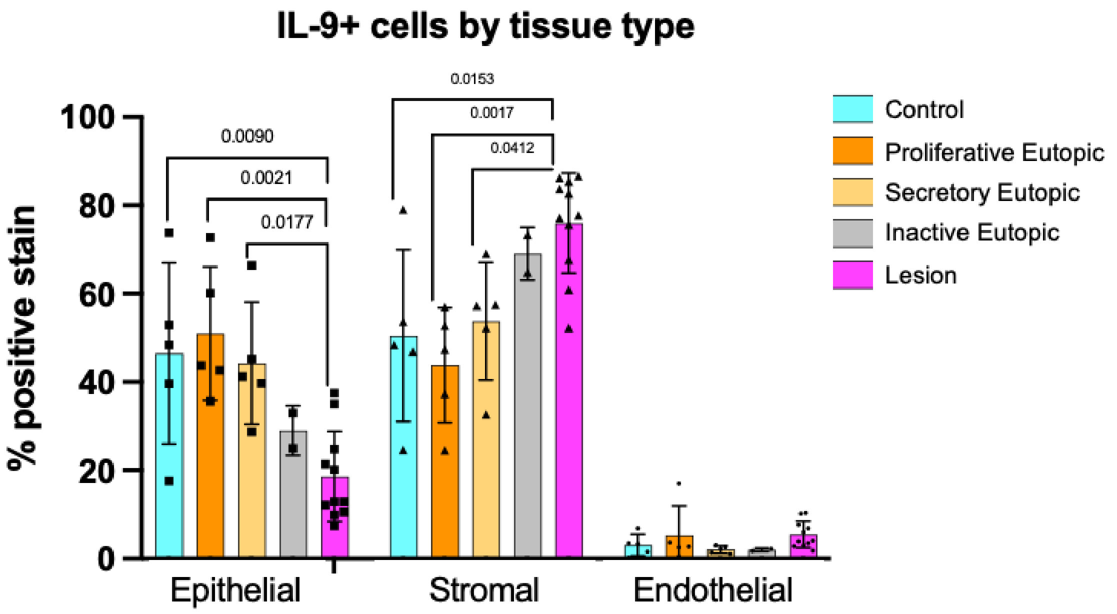

Supplemental figure 1. Stromal proportion of IL-9+ cells is significantly higher in lesion samples, epithelial proportion significantly lower. Out of IL-9+ cells, the portion represented by stromal cells was significantly higher for the lesion sample group (n= 11) as compared to control endometrium (n= 5), proliferative eutopic endometrium (n= 5), and secretory eutopic endometrium (n= 5). Inactive endometrium appears to be most similar to lesion tissue in terms of IL-9+ distribution.

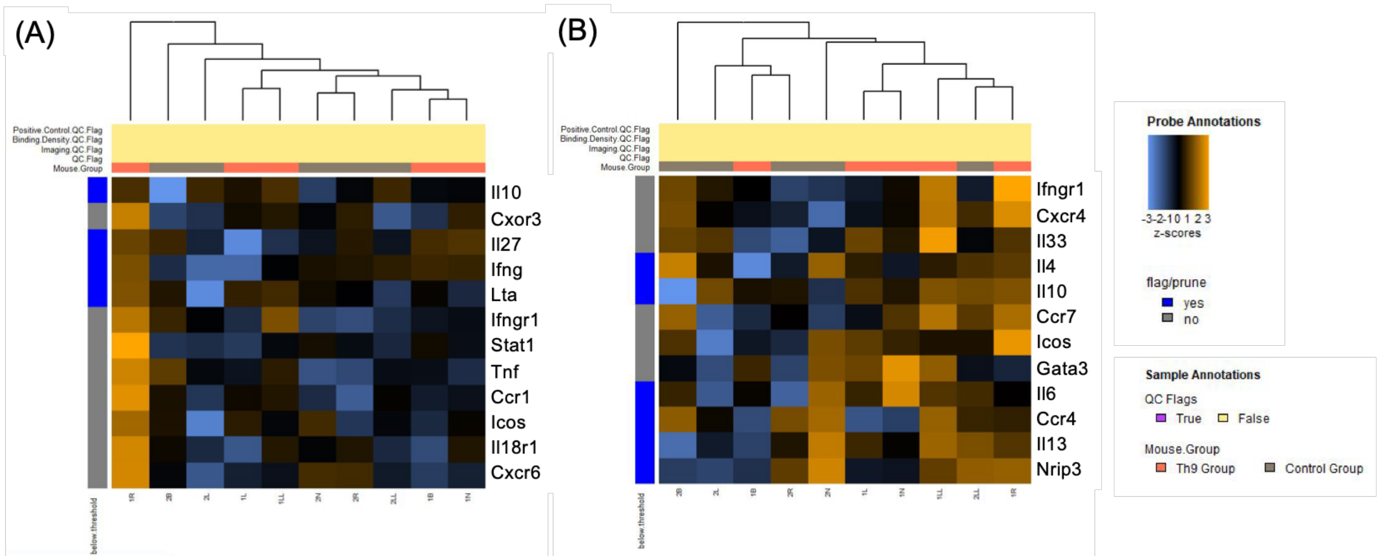

Supplemental figure 2: Unsupervised heatmaps of normalized gene expression data pertaining to (A) M1 and (B) M2 macrophage activation in mouse endometriotic lesions of mice receiving Th9 adoptive transfer (n=5) or PBS (n=5). No clear unsupervised clustering was found when visualizing expression within these murine treatment groups. Orange= increased expression, blue= decreased expression, in accordance with the z-score scale (top right).
